# Effects of short-term radiation emitted by WCDMA mobile phones on teenagers and adults

**DOI:** 10.1186/1471-2458-14-438

**Published:** 2014-05-10

**Authors:** Soo Beom Choi, Min Kyung Kwon, Jai Won Chung, Jee Soo Park, KilSoo Chung, Deok Won Kim

**Affiliations:** 1 Department of Medical Engineering, Yonsei University College of Medicine, Seoul, Republic of Korea; 2 Brain Korea 21 PLUS Project for Medical Science, Yonsei University College of Medicine, Seoul, Republic of Korea; 3 Graduate Program in Biomedical Engineering, Yonsei University, Seoul, Republic of Korea; 4 Department of Medicine, Yonsei University College of Medicine, Seoul, Republic of Korea; 5 Department of Electrical System, Dong Yang Mirae University, Seoul, Republic of Korea

**Keywords:** Physiological changes, Subjective symptoms, RF-EMFs perception, Provocation, ANS, Smart phones, Teenagers

## Abstract

**Background:**

With the rapid increasing use of third generation (3 G) mobile phones, social concerns have arisen concerning the possible health effects of radio frequency-electromagnetic fields (RF-EMFs) emitted by wideband code division multiple access (WCDMA) mobile phones in humans. The number of people, who complain of various symptoms such as headache, dizziness, and fatigue, has also increased. Recently, the importance of researches on teenagers has been on the rise. However, very few provocation studies have examined the health effects of WCDMA mobile phone radiation on teenagers.

**Methods:**

In this double-blind study, two volunteer groups of 26 adults and 26 teenagers were simultaneously investigated by measuring physiological changes in heart rate, respiration rate, and heart rate variability for autonomic nervous system (ANS), eight subjective symptoms, and perception of RF-EMFs during sham and real exposure sessions to verify its effects on adults and teenagers. Experiments were conducted using a dummy phone containing a WCDMA module (average power, 250 mW at 1950 MHz; specific absorption rate, 1.57 W/kg) within a headset placed on the head for 32 min.

**Results:**

Short-term WCDMA RF-EMFs generated no significant changes in ANS, subjective symptoms or the percentages of those who believed they were being exposed in either group.

**Conclusions:**

Considering the analyzed physiological data, the subjective symptoms surveyed, and the percentages of those who believed they were being exposed, 32 min of RF radiation emitted by WCDMA mobile phones demonstrated no effects in either adult or teenager subjects.

## Background

With the increasing use of third generation (3 G) mobile phones, social concerns have arisen concerning the possible health effects of radio frequency-electromagnetic fields (RF-EMFs) emitted by wideband code division multiple access (WCDMA) mobile phones in humans [1]. On the basis of limited evidence from both human and animal studies, the World Health Organization (WHO) has classified RF-EMFs as possibly carcinogenic to humans (Group 2B) [2]. WHO considered the RF-EMFs provocation studies on children of different ages to be a high-priority research in the 2010 Research Agenda [3]. Russian National Committee on Non-Ionizing Radiation Protection (RNCNIRP) announced that absorption of EMF in a child’s brain was greater than in an adult’s brain because larger brain areas including those responsible for intellectual development were exposed in a child’s brain in their resolution [4]. As a child’s brain is also undergoing development and its intellectual functions are maturing, it is more susceptible to environmental hazards than an adult’s brain.

Lindholm et al. [5] monitored local cerebral blood flow during exposure to Global System for Mobile Communication (GSM) mobile phone radiation in a teenager group (14 – 15 years old). They also measured electrocardiogram (ECG), blood pressure, and temperature simultaneously. They concluded that there were no significant changes during the short-term RF-EMFs exposure. Kramarenko and Tan recorded electroencephalogram (EEG) changes during the exposure of ten adults and ten children (12 years old) to a GSM phone. They suggested that cellular phones may reversibly influence the human brain [6]. Preece et al. [7] examined whether a standard mobile phone exposure at 902 MHz had a significant effect on cognitive function in 18 children (10 – 12 years old). There was a tendency for reaction time to be shorter during exposure to radiation than in the sham condition, but no effects reached statistical significance after the Bonferroni correction. Haarala et al. [8] investigated the potential effects of a standard 902 MHz GSM mobile phone on 10 – 14 year old children’s cognitive function, and found that the mobile phone had no effect on children’s cognitive function. Kwon et al. [9] investigated the effects of GSM mobile phone use on the auditory sensory memory in 17 children (11 – 12 years old). They found that a short exposure to mobile phone EMF had no statistically significant effects on the neural change-detection profile measured with mismatch negativity. Although such studies as mentioned above have examined the effects of GSM mobile phone on teenagers or children, there are a few studies investigating about the effects of WCDMA mobile phone radiation on children or teenagers.

The autonomic nervous system (ANS) plays an important role not only in physiological situations, but also in various pathological settings. Among the different available noninvasive techniques for assessing the ANS, heart rate variability (HRV), which is obtained from heart rate, has emerged as a simple and noninvasive method to evaluate the sympathovagal balance at the sinoartrial level [10]. Respiration rate is also closely associated with HRV [11]. Therefore, we selected the three physiological variables including heart rate, respiration rate, and HRV to assess ANS activity.

In this double-blind study, two volunteer groups of 26 adults and 26 teenagers who were mostly middle school students were simultaneously investigated by measuring physiological changes in heart rate, respiration rate, and HRV for ANS, eight subjective symptoms, and perception of RF-EMFs during sham and real exposure sessions. In contrast to many other studies that have examined certain aspects of physiological changes, subjective symptoms, or perception respectively, this study investigated simultaneously these three factors to more reliably examine the bio-effects of WCDMA mobile phone radiation on two groups, especially teenagers. The aim of this study was to test whether RF-EMFs affected heart rate, respiration rate, and HRV, or gave rise to subjective symptoms in adults and teenagers. We also compared the ability of adults and teenagers to perceive exposure to RF radiation. We tested the null hypothesis that adult and teenager groups would have no differences in ANS, subjective symptoms, or perception between sham and real exposures.

## Methods

### Participants

The experiment was performed as a double-blind study with a total of 52 subjects: 26 adults and 26 teenagers. Only healthy subjects without any diseases and not on medications were chosen for the two groups, and 14 – 17 year old subjects were selected for teenager group because the experiment was demanding and potentially stressful, we did not recruit children younger than 14 years old. We used the electromagnetic hypersensitivity (EHS) screening tool developed by Eltiti et al. [12] to exclude EHS subjects. We excluded electromagnetic hypersensitive individuals, because their conditions were more psychological than physiochemical, resulting in some possible bias in our results [13]. Moreover, we already investigated effects of WCDMA mobile phone radiation on electromagnetic hypersensitive subjects [14].

As shown in Table 1, there were no significant differences in male-to-female ratio, height, weight, body-mass index, smoking status, TV viewing time per day (hr), or mobile phone usage time per day (hr) between the two groups. Because of the different characteristics of two groups, there were significant differences in age, computer usage time per day (hr), and mobile phone usage periods (yr).

**Table 1:** Demographics of participants

	**Adult**	**Teenager**	***P*-value**
No. of subjects (n)	26	26	-
Male: female	13: 13	13: 13	0.999
Age (yr)	28.4 ± 5.1	15.3 ± 0.7	< 0.001
Height (cm)	167.1 ± 8.0	164.4 ± 7.3	0.207
Weight (kg)	59.4 ± 11.1	57.8 ± 10.4	0.590
Body mass index (kg/m^2^)	21.1 ± 2.3	21.3 ± 2.8	0.796
Non-smoker: smoker	24 : 2	25 : 1	0.999
Computer usage time (hr/day)	5.3 ± 3.7	2.2 ± 2.0	0.002
TV viewing time (hr/day)	1.7 ± 1.1	1.9 ± 1.6	0.783
Mobile phone usage time (hr/day)	0.6 ± 0.5	1.3 ± 1.3	0.116
Mobile phone usage periods (yr)	11.8 ± 2.4	5.7 ± 1.9	< 0.001

The participants were advised not to consume caffeine, smoke or exercise before the day of the experiment to minimize confounding factors. All subjects, who were recruited by advertisements at the Yonsei University Health System, in Seoul, Korea, were informed of the purpose and procedure of the experiment, and were required to give written consent to participate. The Institutional Review Board of the Yonsei University Health System approved the protocol of this study (project no: 1-2010-0030).

### Experimental setup

The laboratory was used exclusively for this experiment, and all other electrical devices were unplugged except for our instruments to minimize background field levels. Background extremely low frequency (ELF) fields at the head level in the laboratory were measured to ensure that they did not influence the subjects. The average ELF electric and magnetic fields were 1.8 ± 0.0 V/m and 0.02 ± 0.01 μT, respectively, measured using an electric and magnetic field analyzer (EHP-50C; NARDA-STS, Milan, Italy). The average RF field was 0.05 ± 0.00 V/m with a microwave frequency range from 1920 to 1980 MHz, measured using a radiation meter (SRM 3000; NARDA-STS, Pfullingen, Germany). Both the average background ELF and RF-EMFs were negligible.

**Figure 1:**
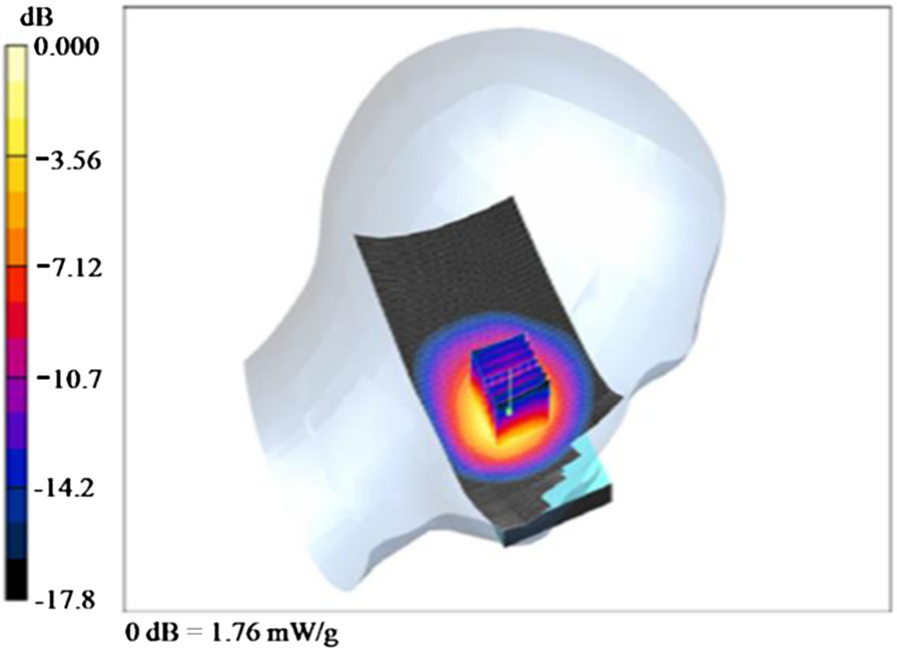
The measured specific absorption rate (SAR) distribution of the wideband code division multiple access (WCDMA) module on the left side.

To achieve better control over exposure, we used a WCDMA module with Qualcomm chipsets (baseband: MSM6290, RF: RFR6285, power management: PM6658, San Diego, CA) to generate WCDMA RF-EMFs instead of a regular smart phone. The WCDMA module continuously transmitted at a mean output power of 250 mW (24 dBm) at 1950 MHz, which was measured using a wireless communication test set (E5515C, Agilent, Santa Clara, CA). The module was inserted into a dummy phone [15], and the location of the module was varied to meet the recommended restriction in specific absorption rate (SAR)_1g_ of 1.6 W/kg for general public, according to the Institute of Electrical and Electronics Engineers (IEEE) Standard [16]. The SAR measurements were made with a DASY 4 measurement system (SPEAG, Zurich, Switzerland), and a Twin SAM (specific anthropomorphic mannequin) phantom was filled with head tissue-equivalent liquid (mass density, 1000 kg/m^3^) as specified by the Federal Communications Commission (FCC). The measured dielectric properties of the liquid were σ = 1.41 S/m and ϵ_r_ = 39.7 for the WCDMA frequency range. When the antenna of the module was positioned 67.5 mm from the ear reference point (ERP) of the dummy phone, the averaged peak spatial SAR_1g_ was determined to be 1.57 W/kg at 1950 MHz at the left cheek position [17]. The electric field and power drift at the ERP were 6.9 V/m and -0.001 dB, respectively. The measured SAR distribution is shown in Figure 1.

**Figure 2:**
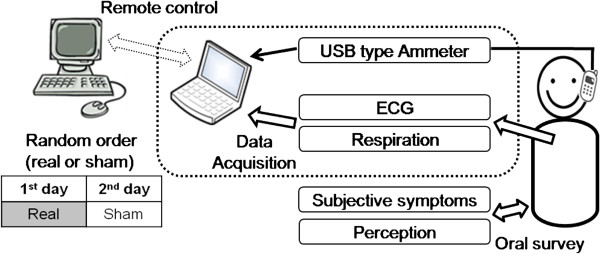
Block diagram of exposure setups.

The module was connected via a 5 m USB cable and a USB type ammeter to a portable laptop computer (X-Note R500, LG Electronics, Seoul, Korea), which controlled the module and monitored electrical current to check exposure conditions (Figure 2). The laptop computer was remotely controlled from another outside desktop computer to satisfy the double-blind study design. The dummy phone was attached to the subject’s head using an earplug and headset to fix it at the ERP next to the cheek [18]. The phone was held at a distance of 3 mm from the ear using a piece of wood for insulation to prevent battery-generated heat from providing subjects with an indication that the phone was working. The apparatus was constructed from only plastic and rubber without any metal [18,19].

### Experimental procedures

No information was given to the subjects except that they would be asked about symptoms and RF-EMFs perception at the beginning of the first experimental day. Sham and real sessions were conducted as a double-blind test to minimize any test bias resulting from a subject and an experimenter recognizing the operational state of the WCDMA module. The experiment was performed for two days, one day for a real session and a second day for a sham session (or vice versa). No matter which came first, sham or real exposure, the second session was always conducted at approximately the same time of the day as the first session in order to maintain the subjects’ physiological rhythm. The order of sham and real sessions for each subject was randomly assigned by our automatic exposure control program using MATLAB 2012b (Mathworks Inc. Natick, MA) to minimize experimental bias. The sham exposure was the first session for 14 teenagers and 15 adults. Time duration between the sessions was a minimum of one day and a maximum of 10 days.

Room temperature and relative humidity, which could considerably affect outcomes, were recorded and maintained as shown in Table 2. For the adult group, room temperature and humidity showed no significant differences between real and sham sessions. For the teenager group, room temperature and humidity showed no significant differences between real and sham sessions. For the sham sessions, room temperature and humidity showed no significant differences between adult and teenager groups. For the real sessions, room temperature and humidity showed no significant differences between adult and teenager groups.

**Table 2:** Room temperature (°C) and relative humidity (%) in the real and sham sessions for the adult and teenager groups (mean ± SD (min-max))

	**Group**	**Real**	**Sham**	***P*-value**
Temperature	Adult	24.5 ± 0.9 (23–26)	24.5 ± 0.7 (23–26)	0.770
	Teenager	24.7 ± 0.9 (23–27)	24.6 ± 0.9 (23–27)	0.731
	*P*-value	0.430	0.724	
Humidity	Adult	40.5 ± 1.9 (37–45)	40.3 ± 3.2 (35–52)	0.823
	Teenager	41.8 ± 2.9 (38–50)	41.5 ± 2.9 (38–50)	0.319
	*P*-value	0.055	0.186	

### Physiological measurements

**Figure 3:**
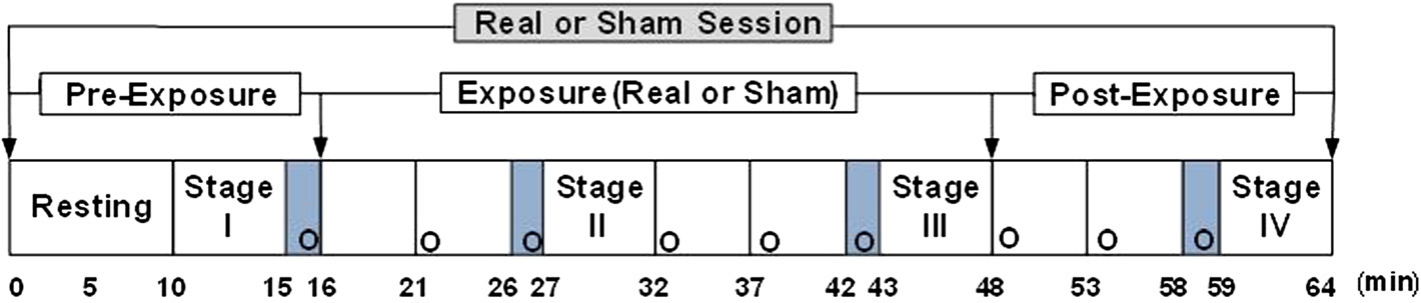
**Experimental procedures for measuring physiological changes and investigating symptoms and perception.** ECG and respiration were measured for 5 min each for four stages. The four shaded areas are periods in which the subjects were questioned about the eight symptoms. “o” indicates timing of the inquiries about RF-EMF perception during each session.

The duration of each exposure session was 64 min, as shown in Figure 3. Before the experiments, subjects were instructed to rest in a sitting position for at least 10 min. Physiological data were collected for 5 min each for four different stages: pre-exposure (stage I), after 11 min of exposure (stage II), after 27 min of exposure (stage III), and post-exposure (stage IV) [14]. At each stage, ECG and respiration were simultaneously measured for 5 min because of the minimum data requirement for HRV [20]. Heart rate, respiration rate, and HRV were obtained with a computerized polygraph (PolyG-I, Laxtha, Daejeon, Korea) with a sampling frequency of 512 Hz. The data were transferred to a laptop computer (X-note R500, LG Electronics, Seoul, Korea) and analyzed using data acquisition software (Telescan 0.9, Laxtha) and analysis software (Complexity software, Laxtha). The PolyG-I recorded ECG through Ag-AgCl electrodes (2223, 3 M, St. Paul, MN) placed on both arms and the right leg of participants.

We first obtained heart rate from ECGs and then acquired HRV and the power spectrum of HRV. High-frequency power (HFP) reflects effects on respiratory sinus arrhythmia, an index of parasympathetic nerve activity, whereas low-frequency power (LFP) reflects effects on both sympathetic and parasympathetic nerves [21]. In this study, the LFP/HFP ratio was used as an index of autonomic nerve activity balance. Respiratory inductance plethysmography, with an excitation frequency of 3 MHz, was used to measure respiration rate. Subjects wore a coiled band around their upper abdomen for measurement of inductance changes resulting from cross-sectional change.

### Subjective symptoms and perception of RF-EMFs

The four shaded areas in Figure 3 denote periods during which subjects were questioned about eight symptoms, with each period lasting approximately 1 min. The eight subjective symptoms of throbbing, itching, warmth, fatigue, headache, dizziness, nausea, and palpitation were evaluated through verbal surveys, which were graded on a 4-point scale ranging from 1 (no sensation) to 4 (strong sensation) as suggested by Koivisto et al. [22]. In addition, perception of EMF exposure was investigated every 5 min throughout the entire session, denoted by an “o” in Figure 3. Subjects were asked to answer the question “Do you believe that you are exposed right now?” nine times during each session. Percentages of those who believed they were being exposed were calculated for pre-exposure, exposure, and post-exposure periods. The total number of inquiries was 260 (5 × 52) during real exposure and 676 (13 × 52) during non-exposure; the total number of subjects was 52 (26 + 26).

### Data analysis

A repeated two-way analysis of variance (ANOVA) was performed using SPSS software (SPSS 20, SPSS, Chicago, IL) to investigate differences in heart rate, respiration rate, and LFP/HFP ratio with exposure and stage for adult and teenager groups. A *P*-value < 0.05 was considered statistically significant. Subjective symptoms, which are ordered paired data, were analyzed using a non-parametric Wilcoxon signed-rank test. A total of 64 *P*-values (4 stages × 8 symptoms × 2 groups) were obtained for the real and sham exposure sessions for the eight symptoms at four stages in both groups. The significance level was adjusted to 0.0125 (0.05/4) because testing was performed in four stages.

There were two exposure sessions for each participant, and nine perception inquiries for each session, as shown in Figure 3. For each session, there was one inquiry during pre-exposure, five inquiries during sham or real exposure, and three inquiries during post-exposure. In both groups, the percentages of those who believed they were being exposed were obtained and evaluated for significant differences between real and sham sessions using McNemar’s test. The pre-exposure period (first inquiry) of the sham sessions was compared with that of the real sessions to test whether conditions before sham and real exposures of subjects were the same. The sham exposure period was compared with the real exposure period to test whether the subjects could detect the fields (second through sixth inquiries). The post-exposure period after sham exposure was compared with the post-exposure period after real exposure to test whether the real exposure influenced the perception of exposure in the post-exposure period (seventh through ninth inquiries).

The significance level of the exposure period was adjusted to 0.01 (0.05/5), and that of the post-exposure period was adjusted to 0.017 (0.05/3) because testing was for five and three inquiries. Fisher’s exact test was applied to evaluate differences in the percentages of those who answered “yes”, which were nominal data, between the adult and teenager groups for sham and real exposure sessions. Fisher’s exact test was used because the expected values in any cells in the contingency table were below 5.

## Results

### Physiological variables

Heart rate, respiration rate, and LFP/HFP ratios of the adult and teenager groups during real and sham exposures are shown in the top section of Table 3. A repeated two-way ANOVA showed no significant differences in heart rate or respiration rate for stage or exposure in either group. However, LFP/HFP ratios showed significant differences by stage in both groups, as shown in the bottom of Table 3. Therefore, a Bonferroni post hoc test was done after two-way ANOVA to investigate any differences in LFP/HFP ratios between stages for each group. For the adult group, LFP/HFP showed no significant difference between real and sham exposures (*P* = 0.307), but did show a significant difference among stages (*P* = 0.033). For the teenager group, LFP/HFP was not significantly different between real and sham exposures (*P* = 0.661), but was significantly different among stages (*P* = 0.002).

**Table 3:** Descriptive and statistical tests for heart rate, respiration rate, and LFP/HFP ratio among stage, exposure, and interaction

	**Heart rate (bpm)**				**Respiration rate (bpm)**				**LFP/HFP ratio**			
	**Adult**		**Teenager**		**Adult**		**Teenager**		**Adult**		**Teenager**	
	**Sham**	**Real**	**Sham**	**Real**	**Sham**	**Real**	**Sham**	**Real**	**Sham**	**Real**	**Sham**	**Real**
Stage	Mean (standard error)											
I	76.6 (2.1)	79.1 (1.9)	79.3 (2.1)	80.9 (1.7)	18.0 (0.5)	18.3 (0.5)	19.3 (0.5)	19.2 (0.4)	1.9 (0.3)	2.3 (0.4)	1.6 (0.3)	1.5 (0.3)
II	76.6 (2.1)	77.9 (1.7)	79.8 (1.8)	80.4 (1.6)	18.2 (0.4)	18.1 (0.6)	19.3 (0.5)	19.3 (0.6)	2.6 (0.4)	3.1 (0.7)	1.7 (0.3)	2.0 (0.4)
III	75.4 (2.0)	77.5 (1.7)	80.7 (1.8)	80.7 (1.7)	18.4 (0.5)	18.2 (0.5)	19.2 (0.5)	19.8 (0.5)	2.3 (0.3)	3.6 (1.0)	2.6 (0.5)	1.9 (0.3)
IV	76.5 (2.1)	77.1 (1.7)	81.2 (1.6)	81.0 (1.7)	18.2 (0.5)	17.9 (0.6)	19.7 (0.5)	20.3 (0.5)	3.2 (0.7)	2.9 (0.6)	2.3 (0.5)	2.3 (0.4)
Factor	*P*-value (F - statistic)											
Exposure	0.328 (0.997)		0.671 (0.184)		0.843 (0.040)		0.433 (0.635)		0.307 (1.088)		0.661 (0.197)	
Stage	0.211 (1.644)		0.323 (1.180)		0.677 (0.510)		0.067 (2.481)		0.033* (3.723)		0.002* (5.492)	
Interaction (exposure and stage)	0.324 (1.168)		0.209 (1.600)		0.633 (0.575)		0.444 (0.903)		0.267 (1.350)		0.222 (1.562)	

*P < 0.05, bpm; beats per min.

LFP/HFP ratio; low-frequency power/high-frequency power (power spectrum of heart rate variability).

Stage I; pre-exposure, Stage II; after 11 min of exposure, Stage III; after 27 min of exposure, Stage IV; post-exposure.

### Subjective symptoms and perception percentages

Neither the adult nor the teenager group showed significant differences in any of the eight subjective symptoms surveyed (throbbing, itching, warmth, fatigue, headache, dizziness, nausea, and palpitation) between sham and real sessions in any of the four stages (Additional file 1: Table S1 and S2).

Table 4 shows the percentages of subjects who believed they were being exposed during exposure (real or sham) in the adult and teenager groups. We compared the percentages of those perceiving exposure during real and sham exposure period (second through sixth inquiries) using McNemar’s test and found no significant difference between real and sham exposure period in the adult or teenager groups. To test for delayed effects of real exposure on post-exposure perception (seventh through ninth inquiries), we applied the same test and found no significant difference in the percentages of those who believed they were being exposed following real and sham exposures in the adult (*P* = 0.999 at all three inquiries) or teenager (*P* = 0.500, *P* = 0.999, *P* = 0.999) groups. Also, no significant difference was seen during pre-exposure period (first inquiry) between real and sham exposures in teenager (*P* = 0.999) group, indicating that the conditions experienced by subjects before real and sham exposures were the same. For adult group, we could not perform McNemar’s test because no one answered “yes” in pre-exposure period. Similarly, a chi-square test for trend showed that the percentages of those who believed they were being exposed during pre-exposure, sham exposure, and post-exposure were not significantly different in the adult (*P* = 0.440) or teenager (*P* = 0.195) groups. This demonstrated that conditions could not be distinguished for participants throughout sham-exposure sessions.

**Table 4:** **percentages of those who believed they were being exposed during sham and real exposure period, and *P*-values for sham and real exposures in adult and teenager groups**

**Group**	**Session**	**Exposure**									
		**2nd**		**3rd**		**4th**		**5th**		**6th**	
		**Mean (%)**	***P*-value**	**Mean (%)**	***P*-value**	**Mean (%)**	***P*-value**	**Mean (%)**	***P*-value**	**Mean (%)**	***P*-value**
Adult (n = 26)	Sham	7.7	0.999	7.7	0.999	3.8	0.999	7.7	0.999	3.8	0.999
	Real	3.8		3.8		7.7		7.7		7.7	
Teenager (n = 26)	Sham	7.7	0.999	0.0	0.250	3.8	0.250	11.5	0.999	7.7	0.999
	Real	7.7		11.5		15.4		11.5		11.5	

**Figure 4:**
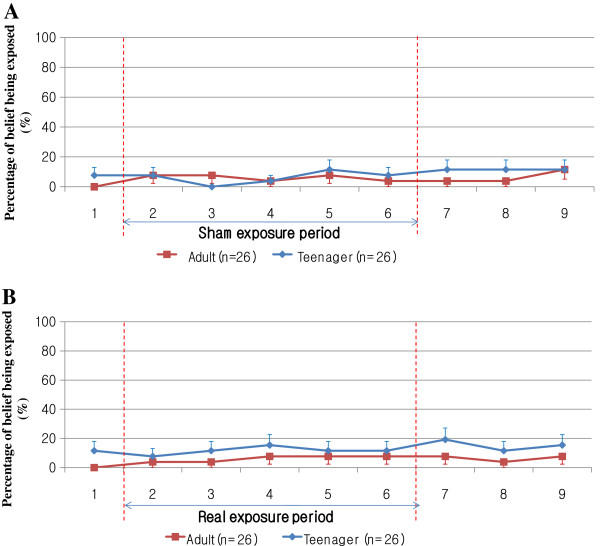
**Percentages who believed they were being exposed at nine inquiry points in adult and teenager groups for sham (A) and real (B) exposure sessions.** Bars indicate standard errors.

Figure 4 shows the percentages of participants in the adult and teenager groups for each inquiry number who believed they were being exposed in sham (Figure 4A) and real (Figure 4B) exposure sessions. No significant differences were seen between the adult and teenager groups in all inquiries during sham or real exposure session. Even though both groups showed low percentages of belief of being exposed during the sham exposure period (Figure 4A), they also showed low percentages during the real exposure period (Figure 4B). In summary, Table 4 shows no significant difference in perception percentages between real and sham exposure period in the adult or teenager groups. Figure 4 also shows no significant difference between the adult and teenager groups in sham or real exposure period. Therefore, we concluded that neither the adult nor the teenager group correctly perceived the RF-EMFs considering Table 4 and Figure 4.

## Discussion

Neither the adults nor the teenagers showed significant differences in heart or respiration rate between real and sham exposures or among stages. For LFP/HFP, however, significant differences were seen between some stages during both real and sham exposure sessions in both groups. One disadvantage of the LFP/HFP analysis is that it is considerably influenced by stress, which can increase or decrease LFP/HFP [23]. Hjortskov et al. [24] reported that psychological stress could result in an increased LFP/HFP. Nam et al. [25] reported that LFP/HFP monotonically increased at each stage during 30 min of sham exposure in both EHS and non-EHS groups. In this experiment, one of the potential sources of stress was the requirement that the subjects not move during the 64-min experiment. In fact, the “no-movement” requirement was the factor that drew the most complaints from the participants, especially the teenagers. Therefore, the significant increase in LFP/HFP with time in the real and sham exposure sessions of both groups must have resulted from factors other than field exposure such as psychological stress, anxiety, or environmental factors.

For the eight subjective symptoms attributed to WCDMA mobile phone radiation, neither the adult group nor the teenager group showed significant differences between sham and real exposures in any of the four stages. Cinel et al. [26] found no evidence suggesting that exposure to mobile phone RF-EMFs affected subjective symptoms. Koivisto et al. [22] also reported that the RF-EMFs exposure did not produce any consistent subjective symptoms or sensations such as headache, dizziness, and fatigue in the non-EHS subjects. In conclusion, RF-EMFs did not give rise to subjective symptoms in adults or teenagers in this study.

No significant differences were seen in the percentages of participants who believed they were being exposed between the real and sham exposures in either the adult or the teenager group. Kwon et al. [27] reported that they found no evidence that their 84 participants perceived GSM mobile phone EMFs. All participants, even including six subjects with high self-rated sensibility, were not able to perceive mobile phone EMFs. No significant differences in percentages of perception were seen for either group among participants who believed they were being exposed during either pre-exposure or post-exposure periods between real and sham exposures. Also, no significant differences were observed in the percentages of perception for either the adult or teenager groups during sham exposure sessions (pre-exposure, sham exposure, post-exposure). Therefore, our experimental protocol appeared to be minimally biased since we confirmed no delayed effects, no differences in pre-exposure condition, and no difference in the percentages of those who believed they were being exposed during the pre-exposure, sham exposure, and post-exposure periods. In this study, the subjects had only two choices, “yes” or “no”, to the perception inquiry of RF-EMFs. However, it could have been biased against subjects who were not sure. For future study, it is recommended to give subjects another choice, “unsure”, and to exclude the answer in calculating the perception accuracy.

Children are more preferable to teenagers as participants in this study because the former are more vulnerable than the latter [28]. However, it is difficult for children due to stress to participate in our experiment, which needs a “no-movement” requirement for approximately one hour. It is also difficult to recruit children because of difficulty in obtaining parents’ approval. We finally recruited teenagers as the second best. Those are the reasons why there are only a few provocation studies with children. Croft et al. [15] measured alpha activity for both GSM and WCDMA exposure among adolescents, young adults, and elderly groups. They reported an effect of GSM exposure in young adults, but observed no effect in adolescents or the elderly, or in any age group, as a function of WCDMA exposure. This result for WCDMA exposure is consistent with ours, even though they examined brain activity and we did heart rate.

There are three limitations in this study. The first limitation is the small number of participants. The number of 26 adults and 26 teenagers may not be to conclude that there are no effects of radiation emitted by WCDMA in both adults and teenagers. Moreover, any effect of WCDMA mobile phone radiation on the autonomic system might be quite limited and difficult to detect. Therefore, to draw some more definitive conclusions on this, a much larger sample will be needed. Secondly, in our study, more subjects received sham exposure for the first session. Ideally, the same number for each session would be better. However, the skewness is small and probably makes no difference. Lastly, we did not investigate the effects of the repetitive and daily regular exposure to RF radiation emitted by WCDMA mobile phones, which could be hazardous to teenagers, as well as adults. Therefore, further study on repetitive and daily regular exposure is necessary to examine the long-term effects, especially on teenagers.

## Conclusions

In both adults and teenagers, there were no significant differences in heart rate, respiration rate, or LFP/HFP, which are all related to ANS, between sham and real exposure to a WCDMA module (average power, 24 dBm at 1950 MHz; specific absorption rate, 1.57 W/kg) for 32 min. There was no association between eight subjective symptoms and short-term RF-EMFs exposure in either group. We could not find evidences of the hypothesis that the self-perception of the exposure between two groups was different. Therefore, based on our physiological data, survey of subjective symptoms, and percentages of participants who believed they were being exposed, no effects were observed in teenagers or adults as a result of 32 min exposure to RF radiation emitted by WCDMA mobile phones.

## Abbreviations

ANOVA: Analysis of variance; ANS: Autonomic nervous system; ECG: Electrocardiogram; EEG: Electroencephalograms; EHS: Electromagnetic hypersensitivity; ELF: Extremely low frequency; EMF: Electromagnetic field; ERP: Ear reference point; FCC: Federal Communications Commission; GSM: Global System for Mobile Communications; HFP: High-frequency power; HRV: Heart rate variability; IEEE: Institute of Electrical and Electronics Engineers; LFP: Low-frequency power; max: Maximum; min: Minimum; n: Number; RF-EMFs: Radio frequency-electromagnetic fields; RNCNIRP: Russian National Committee on Non-Ionizing Radiation Protection; SAR: Specific absorption rate; SD: Standard deviation; WCDMA: Wideband code division multiple access; WHO: World Health Organization; yr: Year; 3 G: Third generation.

## Competing interests

The authors declare that they have no competing interests.

## Authors’ contributions

SBC set up the WCDMA module and collected experimental data. MKK performed statistical analyses, and JWC, JSP and KSC recruited the subjects and collected experimental data. DWK contributed to the development of the study protocol and editing of the manuscript. All authors read and approved the final manuscript.

## Pre-publication history

The pre-publication history for this paper can be accessed here:

http://www.biomedcentral.com/1471-2458/14/438/prepub

## Supplementary Material

Additional file 1: Table S1 Eight subjective symptoms of the each stage for the real and sham sessions in the adult group. **Table S2.** Eight subjective symptoms of the each stage for the real and sham sessions in the teenager group. Click here for file
