# Supplementary material for: Effects of short-term radiation emitted by WCDMA mobile phones on teenagers and adults
Source: BMC Public Health. 2014 May 10;14:438. doi: 10.1186/1471-2458-14-438 (PMC4108016; doi:10.1186/1471-2458-14-438)
Supplement: Additional file 1: Table S1 — Eight subjective symptoms of the each stage for the real and sham sessions in the adult group. Table S2. Eight subjective symptoms of the each stage for the real and sham sessions in the teenager group. [file 1471-2458-14-438-S1.doc]

Table S1. Eight subjective symptoms of the each stage for the real and sham sessions in the adult group

| **symptoms** | **Stage** | **Sham**  **(Mean ± SD)** | **Real**  **(Mean ± SD)** | **P-value** | **Symptoms** | **Stage** | **Sham**  **(Mean ± SD)** | **Real**  **(Mean ± SD)** | **P-value** |
| --- | --- | --- | --- | --- | --- | --- | --- | --- | --- |
| Itching | I | 1.0 ± 0.2 | 1.0 ± 0.0 | 0.317 | Headache | I | 1.0 ± 0.2 | 1.0 ± 0.0 | 0.317 |
|  | II | 1.0 ± 0.2 | 1.0 ± 0.0 | 0.317 |  | II | 1.1 ± 0.3 | 1.0 ± 0.2 | 0.157 |
|  | III | 1.0 ± 0.2 | 1.0 ± 0.0 | 0.317 |  | III | 1.2 ± 0.4 | 1.1 ± 0.3 | 0.564 |
|  | IV | 1.0 ± 0.2 | 1.0 ± 0.0 | 0.317 |  | IV | 1.3 ± 0.5 | 1.3 ± 0.5 | 0.999 |
| Throbbing | I | 1.0 ± 0.0 | 1.0 ± 0.0 | 0.999 | Dizziness | I | 1.1 ± 0.3 | 1.0 ± 0.0 | 0.157 |
|  | II | 1.0 ± 0.0 | 1.0 ± 0.0 | 0.999 |  | II | 1.1 ± 0.3 | 1.0 ± 0.2 | 0.317 |
|  | III | 1.1 ± 0.3 | 1.1 ± 0.3 | 0.317 |  | III | 1.1 ± 0.3 | 1.1 ± 0.3 | 0.999 |
|  | IV | 1.0 ± 0.2 | 1.0 ± 0.2 | 0.999 |  | IV | 1.1 ± 0.4 | 1.2 ± 0.5 | 0.655 |
| Warmth | I | 1.1 ± 0.3 | 1.0 ± 0.2 | 0.564 | Nausea | I | 1.0 ± 0.0 | 1.0 ± 0.0 | 0.999 |
|  | II | 1.1 ± 0.3 | 1.2 ± 0.4 | 0.317 |  | II | 1.0 ± 0.2 | 1.0 ± 0.0 | 0.317 |
|  | III | 1.2 ± 0.4 | 1.2 ± 0.5 | 0.564 |  | III | 1.1 ± 0.4 | 1.0 ± 0.2 | 0.317 |
|  | IV | 1.1 ± 0.3 | 1.2 ± 0.5 | 0.257 |  | IV | 1.1 ± 0.4 | 1.1 ± 0.4 | 0.999 |
| Fatigue | I | 1.0 ± 0.0 | 1.0 ± 0.2 | 0.317 | Palpitation | I | 1.1 ± 0.3 | 1.0 ± 0.2 | 0.564 |
|  | II | 1.0 ± 0.2 | 1.1 ± 0.4 | 0.414 |  | II | 1.0 ± 0.2 | 1.0 ± 0.0 | 0.317 |
|  | III | 1.1 ± 0.3 | 1.2 ± 0.5 | 0.414 |  | III | 1.0 ± 0.0 | 1.0 ± 0.0 | 0.999 |
|  | IV | 1.2 ± 0.7 | 1.2 ± 0.5 | 0.655 |  | IV | 1.0 ± 0.2 | 1.0 ± 0.0 | 0.317 |

P-values were obtained by Wilcoxon signed rank-test.

A significance level of 0.0125 (four stage)

Table S2. Eight subjective symptoms of the each stage for the real and sham sessions in the teenager group

| **symptoms** | **Stage** | **Sham**  **(Mean ± SD)** | **Real**  **(Mean ± SD)** | **P-value** | **Symptoms** | **Stage** | **Sham**  **(Mean ± SD)** | **Real**  **(Mean ± SD)** | **P-value** |
| --- | --- | --- | --- | --- | --- | --- | --- | --- | --- |
| Itching | I | 1.2 ± 0.4 | 1.1 ± 0.3 | 0.317 | Headache | I | 1.0 ± 0.0 | 1.1 ± 0.3 | 0.157 |
|  | II | 1.1 ± 0.3 | 1.0 ± 0.2 | 0.157 |  | II | 1.5 ± 0.4 | 1.2 ± 0.5 | 0.999 |
|  | III | 1.3 ± 0.7 | 1.0 ± 0.2 | 0.059 |  | III | 1.4 ± 0.6 | 1.3 ± 0.5 | 0.480 |
|  | IV | 1.1 ± 0.3 | 1.1 ± 0.3 | 0.564 |  | IV | 1.2 ± 0.5 | 1.4 ± 0.6 | 0.083 |
| Throbbing | I | 1.1 ± 0.3 | 1.0 ± 0.2 | 0.317 | Dizziness | I | 1.1 ± 0.3 | 1.1 ± 0.3 | 0.999 |
|  | II | 1.2 ± 0.5 | 1.1 ± 0.3 | 0.257 |  | II | 1.2 ± 0.4 | 1.2 ± 0.5 | 0.564 |
|  | III | 1.2 ± 0.6 | 1.2 ± 0.4 | 0.480 |  | III | 1.2 ± 0.5 | 1.2 ± 0.5 | 0.655 |
|  | IV | 1.3 ± 0.5 | 1.2 ± 0.4 | 0.480 |  | IV | 1.2 ± 0.5 | 1.3 ± 0.5 | 0.655 |
| Warmth | I | 1.0 ± 0.2 | 1.2 ± 0.4 | 0.083 | Nausea | I | 1.0 ± 0.0 | 1.0 ± 0.0 | 0.999 |
|  | II | 1.1 ± 0.3 | 1.2 ± 0.4 | 0.655 |  | II | 1.0 ± 0.0 | 1.0 ± 0.0 | 0.999 |
|  | III | 1.2 ± 0.4 | 1.2 ± 0.5 | 0.317 |  | III | 1.0 ± 0.2 | 1.1 ± 0.3 | 0.317 |
|  | IV | 1.2 ± 0.4 | 1.2 ± 0.5 | 0.414 |  | IV | 1.1 ± 0.3 | 1.1 ± 0.3 | 0.564 |
| Fatigue | I | 1.2 ± 0.4 | 1.4 ± 0.6 | 0.166 | Palpitation | I | 1.0 ± 0.2 | 1.1 ± 0.3 | 0.317 |
|  | II | 1.4 ± 0.5 | 1.3 ± 0.5 | 0.655 |  | II | 1.0 ± 0.2 | 1.1 ± 0.3 | 0.317 |
|  | III | 1.5 ± 0.8 | 1.6 ± 0.7 | 0.593 |  | III | 1.1 ± 0.3 | 1.2 ± 0.4 | 0.157 |
|  | IV | 1.5 ± 0.7 | 1.7 ± 0.6 | 0.157 |  | IV | 1.1 ± 0.3 | 1.1 ± 0.3 | 0.317 |

P-values were obtained by Wilcoxon signed rank-test.

A significance level of 0.0125 (four stage)
